# A real-world data analysis of piroxicam in the FDA Adverse Event Reporting System (FAERS) database

**DOI:** 10.3389/fmed.2025.1687088

**Published:** 2025-10-10

**Authors:** Yanhe Wang, Qiuhong Kong

**Affiliations:** ^1^Department of Orthopedics and Traumatology, Tai’an Traditional Chinese Medicine Hospital, Tai’an, Shandong, China; ^2^Department of Emergency, Jinan Central Hospital, Jinan, Shandong, China

**Keywords:** piroxicam, FAERS, disproportionality analysis, adverse event, real-world safety

## Abstract

**Background:**

Piroxicam is a widely used antipyretic and analgesic. Due to an increasing number of adverse event (AE) reports, effective pharmacovigilance is essential for evaluating its benefit-risk profile.

**Methods:**

We assessed the safety profile of piroxicam through disproportionality analysis based on all AE reports involving the drug in the FDA Adverse Event Reporting System (FAERS) from 2004 till 2024. Signal detection was performed using the reporting odds ratio, proportional reporting ratio, multi-item gamma Poisson shrinker, and Bayesian confidence propagation neural network methods. The Weibull distribution was applied to model time-to-onset of AEs. Analyses were stratified by age and sex, and subgroup patterns were examined. AE outcomes were categorized accordingly.

**Results:**

Our findings confirmed known label-reported AEs, such as hypersensitivity, urticaria, gastric ulcers, and gastrointestinal hemorrhage. Additionally, several potentially less well-known AEs were identified, such as acute generalized exanthematous pustulosis, blister formation, and urinary retention. Subgroup analyses revealed significant variations in AE patterns across different age groups and sexes. The majority of AEs occurred during the early stages of treatment, highlighting the importance of vigilant monitoring of AEs especially during initial dosing.

**Conclusion:**

This real-world study reinforces established safety concerns related to piroxicam while identifying potentially less well-known safety signals. These findings offer valuable insights for clinicians aiming to optimize patient safety during piroxicam therapy.

## 1 Introduction

Piroxicam is a non-steroidal anti-inflammatory drug (NSAID) belonging to the oxicam class, commonly prescribed for managing acute pain and chronic inflammatory diseases such as osteoarthritis and rheumatoid arthritis due to its analgesic, anti-inflammatory, and antipyretic properties (1). Arthritis, particularly its osteoarthritis and rheumatoid arthritis forms, presents a significant public health challenge, affecting the quality of life and daily functioning (2, 3). Chronic pain associated with arthritis can lead to physical limitations and psychological distress, including depression and anxiety (4). The social burden of arthritis-induced pain is substantial, with implications that extend beyond individual discomfort.

Piroxicam exerts its effects by inhibiting cyclooxygenase enzymes (COX-1 and COX-2), thereby reducing in the synthesis of prostaglandins key mediators of pain and inflammation (5). A notable advantage of piroxicam is its long half-life, which allows once-daily dosing, thereby improving patient compliance compared with other NSAIDs requiring multiple doses throughout the day (6). It has also demonstrated efficacy in providing pre-emptive analgesia, significantly reducing postoperative pain and minimizing the need for additional analgesics (7). However, its safety profile warrants careful consideration. Common side effects include gastrointestinal disturbances such as nausea, vomiting, dyspepsia, and more severe complications like gastric ulcers or bleeding due to COX-1 inhibition (8). Long-term use of piroxicam has also been linked to an increased risk of cardiovascular events, particularly in patients with pre-existing heart diseases (9).

The FDA Adverse Event Reporting System (FAERS) is a database created by the U.S. Food and Drug Administration (FDA) to collect and evaluate reports on drug-related adverse events (AEs) (10). This vital pharmacovigilance tool thus aids the U.S. FDA in recognizing possible drug-related safety issues. By analyzing FAERS data, this study assessed the real-world safety of piroxicam, offering healthcare professionals crucial information for making informed prescribing decisions.

## 2 Materials and methods

### 2.1 Data sources, management, and study design

This research used raw data sourced from the FAERS database–a non-mandatory, publicly accessible reporting system that primarily receives submissions from consumers, pharmacists, physicians, and various other stakeholders. The analysis included all reports of AEs in the original ASCII data packet format, wherein piroxicam was recognized as the main drug of concern, covering the timeframe from the first quarter of 2004 through the fourth quarter of 2024.

**FIGURE 1:**
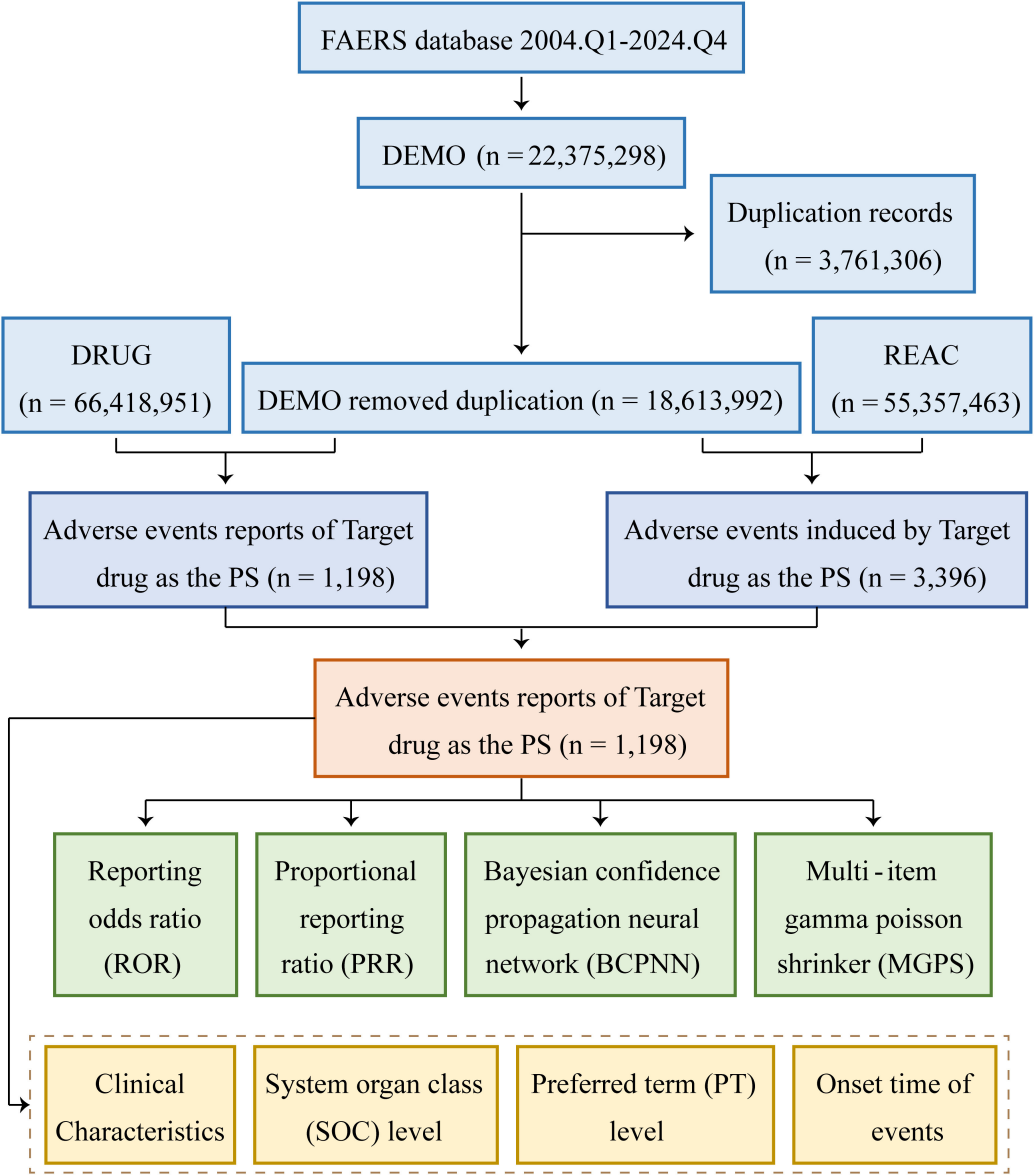
Flowchart demonstrating the AE analysis process for piroxicam using the FAERS database. DEMO, demographics; REAC, reaction; PS, primary suspect.

In terms of data management, this process primarily focused on the removal of duplicate reports and the standardization of AE terminology. The approach for addressing duplicate reports followed the procedures recommended by the FDA. For reports sharing the same case identifier (CASEID), the report with the most recent FDA receipt date (FDA_DT) was preserved. In cases where the CASEID and FDA_DT values were identical, reports possessing the largest unique identifier (PRIMARYID) were retained. In addition, the MedDRA dictionary 27.1 was used to standardize AE terminology, which included preferred terms (PTs) and the corresponding system organ classifications (SOC), so as to improve the reliability of subsequent statistical analysis. A comprehensive flowchart outlining the study design has been presented in Figure 1.

### 2.2 Statistical analysis

A descriptive analysis was performed to outline the characteristics of AE reports linked to piroxicam. Four methods for disproportionate analysis were thereby applied to identify signals indicating potential adverse reactions to piroxicam. These methods included the reporting odds ratio (ROR) (11), proportional reporting ratio (PRR) (12), Bayesian confidence propagation neural network (BCPNN) (13), and multi-item gamma Poisson shrinker (MGPS) (14). An AE was deemed a potential reaction if it surpassed the positive threshold in at least one of these methods (10). Supplementary Table 1 shows the detailed two-by-two contingency tables, whereas Supplementary Table 2 lists the formulas and thresholds used for various disproportionate analyses.

The onset timing of AEs connected to piroxicam was characterized based on the period between the occurrence of reported AEs [as sourced from the DEMO (Demonstration) file] and the initiation of Piroxicam treatment [derived from the THER file (Therapeutic Period File)]. In order to simulate the temporal fluctuations in the occurrence rate of AEs, Weibull distribution was employed. The Weibull distribution was selected primarily due to its superior flexibility in characterizing time-to-event data–a critical advantage over the exponential and log-normal distributions–especially in capturing the dynamic hazard patterns of drug-induced AEs. All analyses were conducted using SAS software version 9.4.

## 3 Results

### 3.1 Clinical characteristics

**FIGURE 2:**
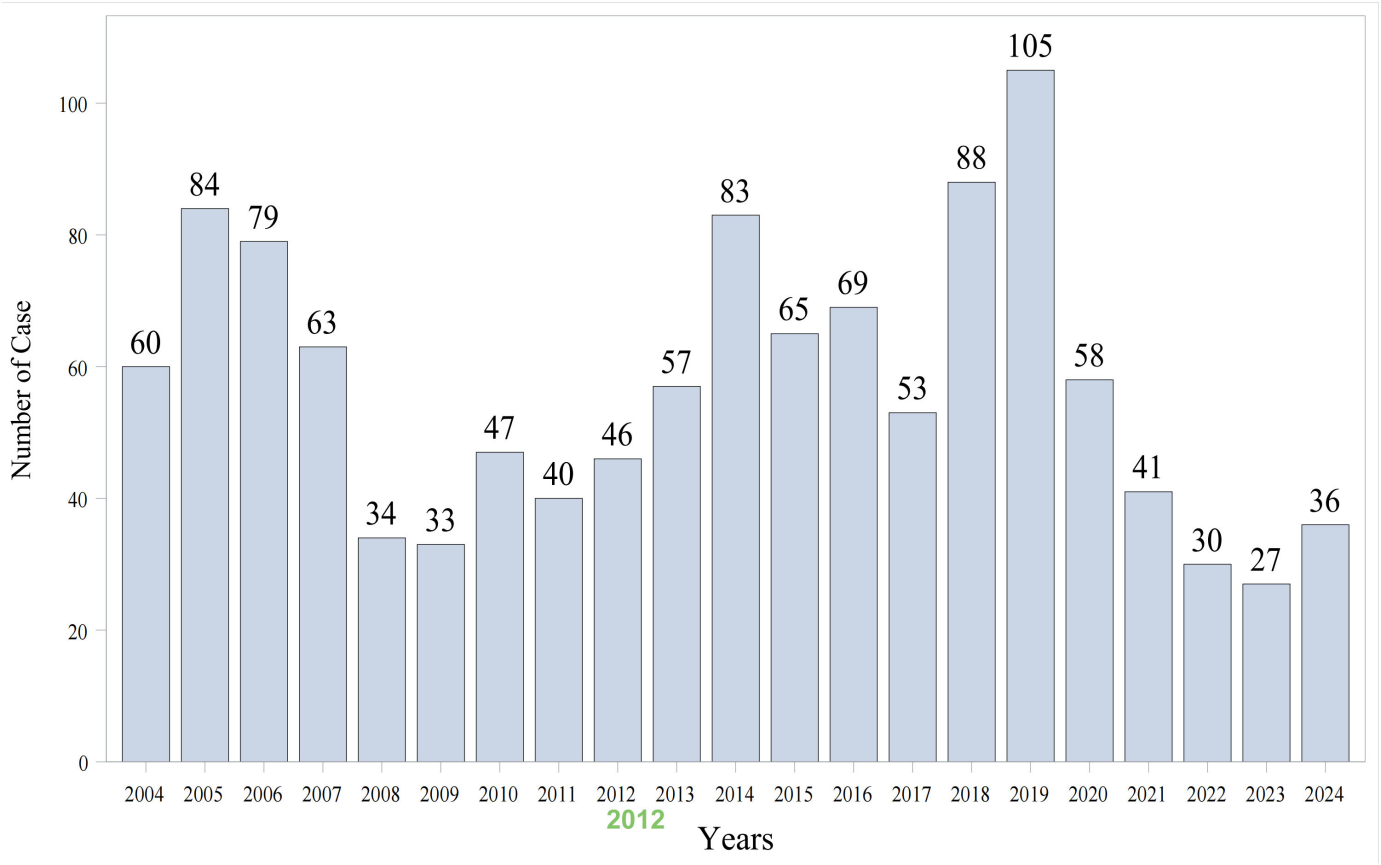
Reports number and trends of piroxicam-related AEs.

As shown in Figure 1, data from 84 quarters (Q1 2004 to Q4 2024) included 18,613,992 patients and 55,357,463 AEs. Piroxicam was listed as the primary suspect drug in 1,198 cases, corresponding to 3,396 AEs. Table 1 presents the clinical characteristics of piroxicam-related events. Of these cases, 60% (*n* = 715) were female patients, while 32% (*n* = 382) were male patients. The majority were aged ≥65 years (28%, *n* = 340), followed by those aged 45–64 years (26%, *n* = 307). Most reports were submitted by physicians (35%, *n* = 418), followed by consumers (25%, *n* = 301) and other healthcare professionals (19%, *n* = 224). Geographically, the majority of reports originated from the United States (43%, *n* = 510), France (15%, *n* = 174), and Brazil (8%, *n* = 91). In terms of outcomes, 29% (*n* = 353) of cases were hospitalized or had prolonged hospital stay. AE reporting showed a fluctuating pattern over the years, peaking in 2018 (7%, *n* = 88) and 2019 (9%, *n* = 105) (Figure 2).

**TABLE 1:** Clinical characteristics of AE reports related to piroxicam from the FAERS database (Q1 2004–Q4 2024).

Characteristics	Number of cases	Proportion of cases (%)
Number of AE reports	1198	
Number of AEs induced by piroxicam	3396	
**Sex**		
Female	715	59.68
Male	382	31.89
Not specified	101	8.43
**Age**		
<18	16	1.34
18–44	179	14.94
45–64	307	25.63
≥65	340	28.38
Not specified	356	29.72
**Reporter**		
Consumer	301	25.13
Lawyer	5	0.42
Not specified	88	7.35
Other health-professional	224	18.70
Pharmacist	162	13.52
Physician	418	34.89
**Top 5 reporting countries**		
United States of America	510	42.57
France	174	14.52
Brazil	91	7.60
Italy	69	5.76
The United Kingdom	56	4.67
**Outcome**		
Life-threatening	57	4.76
Hospitalization - initial or prolonged	353	29.47
Disability	41	3.42
Death	69	5.76
Other serious	498	41.57

Life-threatening: refers to reports that explicitly indicate the event posed an immediate risk of death to the patient at the time of occurrence. For example, reports described as “anaphylactic shock.” Other serious: refers to events that do not meet other specific serious criteria (such as death, hospitalization, etc.), but could lead to serious health consequences if not managed. For example, reports described as “severe drug rash” and “urinary retention.”

### 3.2 Distribution of AEs at the SOC level

**FIGURE 3:**
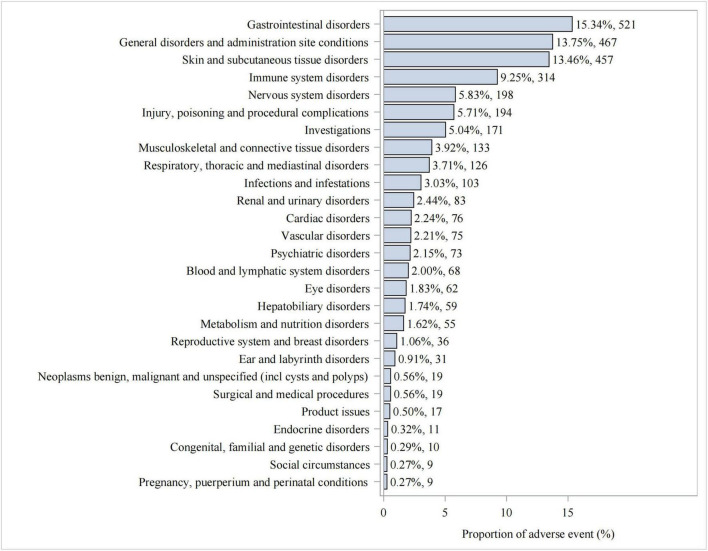
Proportion of AEs by system organ classification (SOC) for piroxicam.

We statistically identified 27 system organ classes associated with piroxicam-induced AEs, suggesting the wide-ranging effects of piroxicam. As shown in Table 2, the strongest signal emerged in immune system disorders (SOC: 10021428, *n* = 314), while gastrointestinal disorders (SOC: 10017947, *n* = 521) were the most frequently reported. Significant AEs were also noted in skin and subcutaneous tissue disorders (SOC: 10040785, *n* = 457) and ear and labyrinth disorders (SOC: 10013993, *n* = 31). Additionally, hepatobiliary disorders (SOC: 10019805, *n* = 59); endocrine disorders (SOC: 10014698, *n* = 11); and respiratory, thoracic, and mediastinal disorders (SOC: 10038738, *n* = 126) were noteworthy. The latter, in particular, represents a less well-known AE not documented in the current piroxicam label information. The distribution of AEs at the SOC level is presented in Figure 3.

**TABLE 2:** Signal intensity of piroxicam-related AEs at the system organ classification (SOC) level in the FAERS database.

System organ class (SOC)	Case reports	ROR (95% CI)	PRR (95% CI)	PRR (χ^2^)	IC (IC025)	EBGM (EBGM05)
Gastrointestinal disorders	521	1.95 (1.78, 2.14)	1.81 (1.67, 1.95)	204.44	0.85 (0.71)	1.81 (1.64)
General disorders and administration site conditions	467	0.75 (0.68, 0.83)	0.79 (0.72, 0.86)	32.1	−0.34 (−0.48)	0.79 (0.72)
Skin and subcutaneous tissue disorders*	457	2.73 (2.47, 3.01)	2.50 (2.29, 2.72)	432.69	1.32 (1.17)	2.49 (2.26)
Immune system disorders*	314	9.14 (8.14, 10.27)	8.39 (7.55, 9.32)	2065.74	3.07 (2.86)	8.39 (7.47)
Nervous system disorders	198	0.67 (0.58, 0.77)	0.69 (0.60, 0.79)	30.68	−0.54 (−0.75)	0.69 (0.60)
Injury, poisoning and procedural complications	194	0.52 (0.45, 0.60)	0.55 (0.48, 0.63)	80.12	−0.86 (−1.07)	0.55 (0.48)
Investigations	171	0.81 (0.70, 0.95)	0.82 (0.71, 0.95)	7.15	−0.29 (−0.51)	0.82 (0.70)
Musculoskeletal and connective tissue disorders	133	0.75 (0.63, 0.89)	0.76 (0.64, 0.89)	10.92	−0.40 (−0.65)	0.76 (0.64)
Respiratory, thoracic and mediastinal disorders	126	0.78 (0.65, 0.93)	0.79 (0.66, 0.94)	7.51	−0.34 (−0.60)	0.79 (0.66)
Infections and infestations	103	0.56 (0.46, 0.69)	0.58 (0.48, 0.70)	33.55	−0.79 (−1.07)	0.58 (0.47)
Renal and urinary disorders	83	1.29 (1.04, 1.61)	1.28 (1.04, 1.59)	5.34	0.36 (0.04)	1.28 (1.03)
Cardiac disorders	76	0.85 (0.68, 1.07)	0.85 (0.68, 1.06)	2	−0.23 (−0.56)	0.85 (0.68)
Vascular disorders	75	1.04 (0.82, 1.30)	1.04 (0.83, 1.30)	0.1	0.05 (−0.28)	1.04 (0.82)
Psychiatric disorders	73	0.37 (0.29, 0.46)	0.38 (0.30, 0.48)	77.27	−1.39 (−1.72)	0.38 (0.30)
Blood and lymphatic system disorders	68	1.19 (0.94, 1.51)	1.19 (0.94, 1.50)	2.02	0.25 (−0.11)	1.19 (0.93)
Eye disorders	62	0.91 (0.71, 1.18)	0.92 (0.72, 1.17)	0.49	−0.13 (−0.49)	0.92 (0.71)
Hepatobiliary disorders	59	1.91 (1.48, 2.47)	1.89 (1.47, 2.44)	25.09	0.92 (0.52)	1.89 (1.46)
Metabolism and nutrition disorders	55	0.74 (0.57, 0.97)	0.75 (0.57, 0.97)	4.82	−0.42 (−0.80)	0.75 (0.57)
Reproductive system and breast disorders	36	1.20 (0.86, 1.66)	1.19 (0.86, 1.65)	1.15	0.26 (−0.23)	1.19 (0.86)
Ear and labyrinth disorders	31	2.12 (1.49, 3.01)	2.11 (1.48, 2.99)	18.09	1.07 (0.51)	2.11 (1.48)
Surgical and medical procedures	19	0.41 (0.26, 0.64)	0.41 (0.26, 0.65)	15.97	−1.27 (−1.88)	0.41 (0.26)
Neoplasms benign, malignant and unspecified (incl cysts and polyps)	19	0.21 (0.13, 0.33)	0.21 (0.14, 0.33)	56.53	−2.23 (−2.82)	0.21 (0.14)
Product issues	17	0.30 (0.19, 0.49)	0.31 (0.19, 0.49)	26.86	−1.70 (−2.32)	0.31 (0.19)
Endocrine disorders	11	1.27 (0.70, 2.30)	1.27 (0.70, 2.29)	0.64	0.35 (−0.52)	1.27 (0.70)
Congenital, familial and genetic disorders	10	0.98 (0.53, 1.82)	0.98 (0.53, 1.82)	0	−0.03 (−0.90)	0.98 (0.53)
Pregnancy, puerperium and perinatal conditions	9	0.62 (0.32, 1.19)	0.62 (0.32, 1.19)	2.12	−0.69 (−1.55)	0.62 (0.32)
Social circumstances	9	0.57 (0.30, 1.09)	0.57 (0.30, 1.09)	2.94	−0.81 (−1.66)	0.57 (0.30)

*Signals were detected when all of the following criteria were met: PRR ≥ 2 and χ^2^ > 4, lower limit of 95% CI of ROR > 1, IC025 > 0, EBGM05 > 2. CI, confidence intervals; EBGM, empirical Bayesian geometric mean; IC, information component.

### 3.3 Distribution of AEs at the PT level

Table 3 lists the 25 most commonly reported PTs. Known side effects such as hypersensitivity, gastric ulcer, urticaria, edema, and eosinophilia were frequently reported. Severe or rare adverse reactions included gastrointestinal hemorrhage, Stevens–Johnson syndrome, and photosensitivity. Unlabeled AEs such as acute generalized exanthematous pustulosis (AGEP), blister formation, and urinary retention also appeared, suggesting potential new safety concerns. A comprehensive list of all reported PT-level AEs is provided in Supplementary Table 3. Table 4 outlines the clinical significance of typical and atypical AEs of piroxicam.

**TABLE 3:** The 25 most common AEs of piroxicam at the PT level.

Preferred term (PT)	Case reports	ROR (95% CI)	PRR (χ^2^)	IC (IC025)	EBGM (EBGM05)
Drug hypersensitivity	249	24.72 (21.73–28.13)	22.99 (5245.55)	4.52 (4.21)	22.95 (20.17)
Gastrointestinal hemorrhage	41	8.58 (6.31–11.68)	8.49 (271.22)	3.09 (2.40)	8.49 (6.24)
Drug interaction	38	4.38 (3.18–6.03)	4.34 (97.84)	2.12 (1.53)	4.34 (3.15)
Urticaria	36	4.07 (2.93–5.65)	4.04 (82.48)	2.01 (1.42)	4.04 (2.91)
Hypersensitivity	33	3.28 (2.33–4.63)	3.26 (51.91)	1.71 (1.11)	3.26 (2.31)
Acute generalized exanthematous pustulosis	27	64.73 (44.29–94.60)	64.22 (1673.95)	6.00 (3.75)	63.97 (43.77)
Melena	24	19.46 (13.02–29.08)	19.33 (416.86)	4.27 (2.90)	19.31 (12.92)
Gastric ulcer	23	21.68 (14.38–32.68)	21.54 (450.05)	4.43 (2.94)	21.51 (14.27)
Fixed eruption	21	223.07 (144.83–343.57)	221.69 (4551.89)	7.77 (3.71)	218.73 (142.01)
Duodenal ulcer	20	51.53 (33.18–80.03)	51.23 (982.08)	5.67 (3.28)	51.08 (32.89)
Blister	17	5.72 (3.55–9.21)	5.70 (65.84)	2.51 (1.49)	5.69 (3.53)
Hematemesis	16	11.28 (6.90–18.44)	11.24 (149.15)	3.49 (2.11)	11.23 (6.87)
Swelling face	16	4.49 (2.75–7.34)	4.48 (43.22)	2.16 (1.19)	4.47 (2.74)
Drug reaction with eosinophilia and systemic symptoms	15	9.78 (5.89–16.24)	9.74 (117.60)	3.28 (1.93)	9.73 (5.86)
Stevens-Johnson syndrome	14	11.25 (6.65–19.01)	11.20 (130.05)	3.48 (1.99)	11.20 (6.62)
Feces discolored	13	10.79 (6.26–18.60)	10.75 (114.92)	3.43 (1.89)	10.74 (6.23)
Gastritis	13	8.81 (5.11–15.18)	8.78 (89.57)	3.13 (1.72)	8.77 (5.09)
Edema	13	4.38 (2.54–7.55)	4.36 (33.73)	2.13 (1.04)	4.36 (2.53)
Face edema	12	12.62 (7.16–22.25)	12.58 (127.84)	3.65 (1.93)	12.57 (7.13)
Photosensitivity reaction	12	13.18 (7.47–23.23)	13.14 (134.46)	3.71 (1.96)	13.13 (7.44)
Toxic epidermal necrolysis	12	14.80 (8.40–26.10)	14.75 (153.76)	3.88 (2.04)	14.74 (8.36)
Dermatitis bullous	11	28.02 (15.49–50.66)	27.93 (285.15)	4.80 (2.27)	27.88 (15.42)
Rectal hemorrhage	10	4.16 (2.24–7.75)	4.16 (23.97)	2.05 (0.82)	4.15 (2.23)
Cross sensitivity reaction	9	90.01 (46.71–173.46)	89.78 (785.80)	6.48 (2.27)	89.29 (46.34)
Urinary retention	9	4.93 (2.56–9.48)	4.92 (28.07)	2.30 (0.91)	4.91 (2.55)

**TABLE 4:** Typical and atypical AEs of piroxicam and their clinical significance.

Adverse effect type	Classic adverse effects	Non-classic adverse effects	Clinical implications
Gastrointestinal	Gastric ulcer	Rectal hemorrhage	Use in conjunction with gastroprotective drugs (such as PPI)
	Gastrointestinal pain	Hematemesis	Monitoring the risk of occult bleeding
Skin	Urticaria	Acute generalized exanthematous pustulosis	Immediately discontinue medication and assess severe skin reactions
	Fixed eruption		Non-typical reactions require identification of drug-related factors
Immune system	Drug hypersensitivity	Cross sensitivity reaction	Inquire about allergy history
		Anaphylactic shock	Emergency measures should be taken in case of severe allergies
General	edema	Swelling face	Distinguish the cause of edema
		Face edema	Isolated facial edema suggests a risk of vascular and neurological edema
Urinary		Urinary retention	Need to monitor urinary function
			May be related to prostaglandin inhibition
Other	Stevens-Johnson syndrome	Toxic epidermal necrolysis	Severe skin and mucosal damage requiring urgent treatment
			Atypical cases need to be reported to the drug surveillance system

### 3.4 Onset time of AEs

**FIGURE 4:**
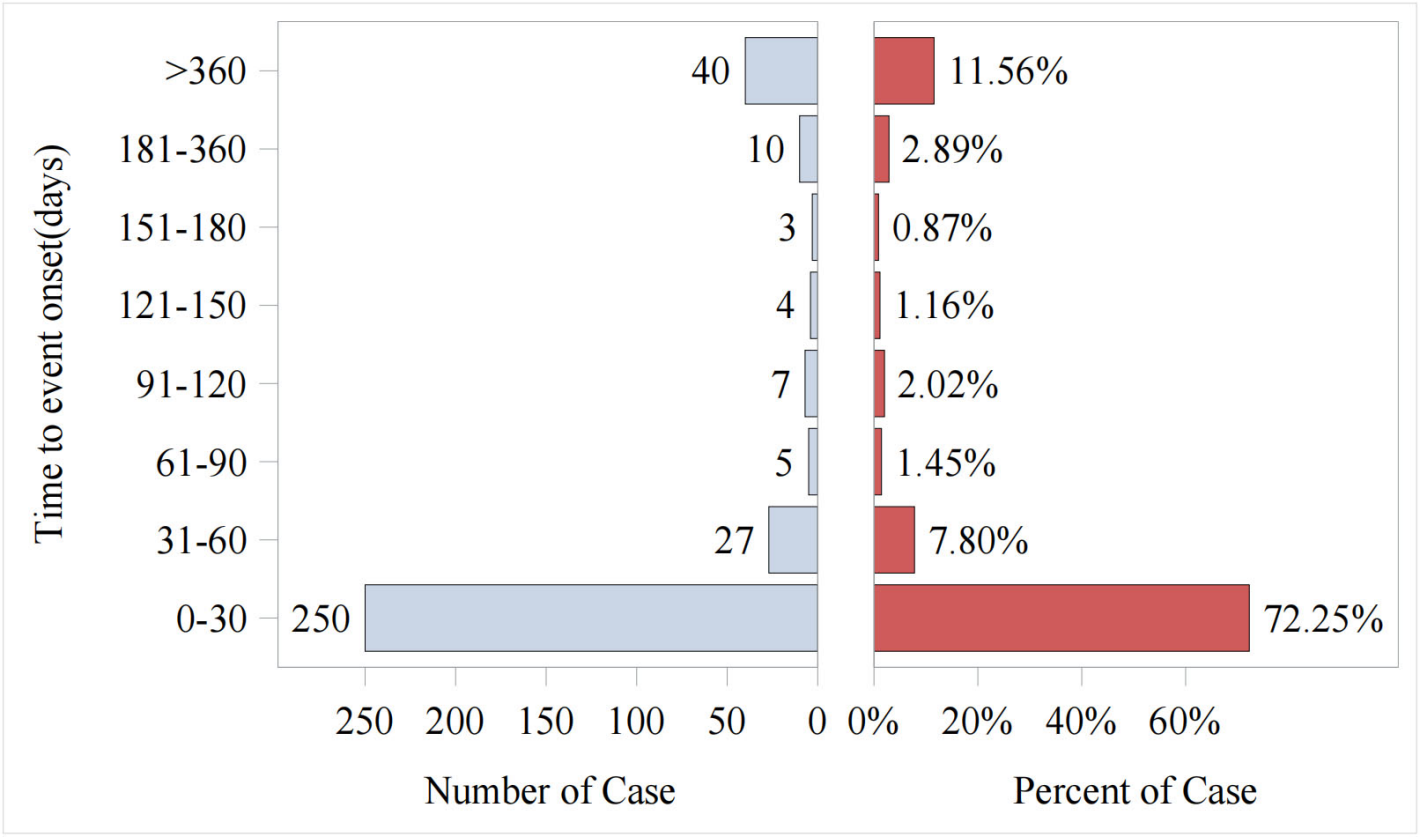
Time to event report distribution of AE reports.

**FIGURE 5:**
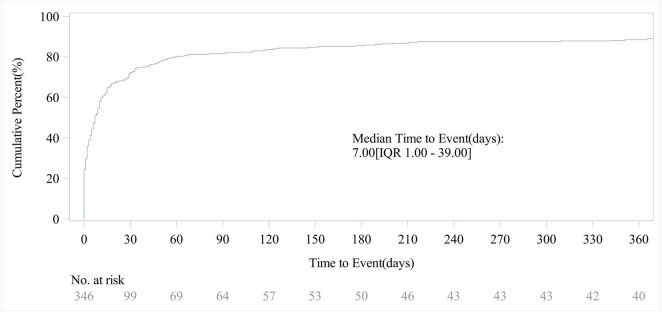
Cumulative incidence of AEs.

Out of the total reports on piroxicam-related AEs, 346 included data on time to onset. As shown in Figure 4, the vast majority of AEs occurred within the first month of piroxicam use (72%, *n* = 250). The cumulative incidence curve for AEs (Figure 5) revealed a median AE onset time of 7 days [interquartile range (IQR) 1–39 days], the shape parameter is 0.4 (95% confidence interval [CI] 0.37–0.44) and the scale parameter is 79 (95% CI 57–109), emphasizing the critical importance of early monitoring during therapy.

### 3.5 Subgroup analysis

Subgroup analysis of AEs associated with piroxicam revealed distinct patterns based on sex and age. Among the 10 most common AEs that met the criteria for a positive signal, female-specific AEs included pruritus, hypersensitivity, erythema, weight loss, and anemia. In contrast, male-specific AEs included melena, AGEP, duodenal ulcer, acute kidney injury (AKI), and fixed drug eruption (Supplementary Figure 1). In patients aged >65 years, frequently reported AEs with positive signals included drug hypersensitivity, gastrointestinal hemorrhage, AKI, melena, and anemia. In the 45–64 years age group, the most common AEs were drug hypersensitivity, rash, urticaria, gastrointestinal hemorrhage, and duodenal ulcer. Among patients aged 18–44 years, pruritus, AGEP, and rash were most prevalent. Notably, two AEs, eye pain and blurred vision, were reported in patients under 18 years (Supplementary Figure 2). The median time to AE onset was 7 days for both sexes, with no significant difference in cumulative incidence curves (Supplementary Figure 3).

### 3.6 Outcome analysis

**FIGURE 6:**
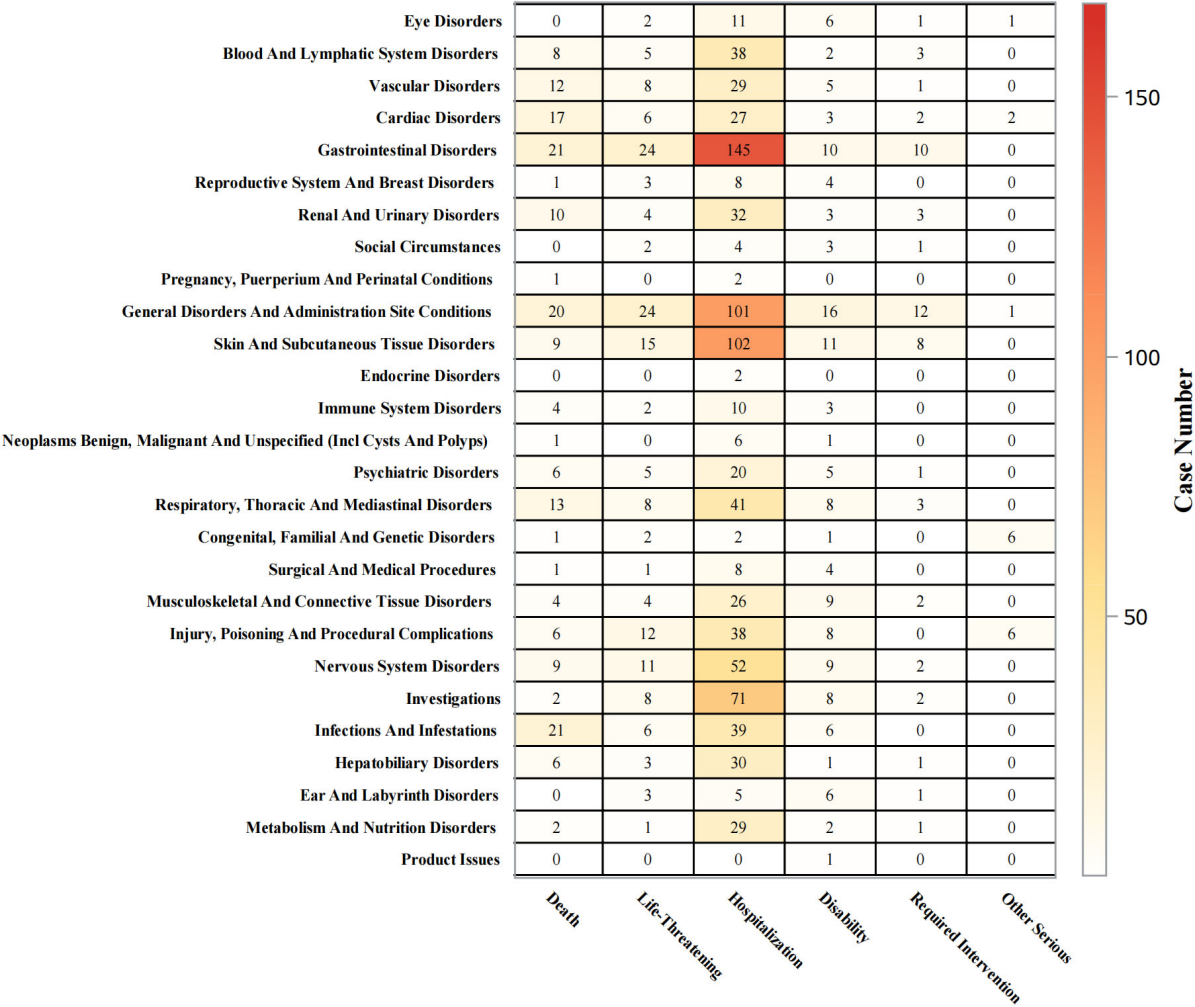
Outcomes by SOCs.

Outcomes of piroxicam-related AEs were statistically analyzed at both SOC and PT levels. As shown in Figure 6, for gastrointestinal disorders, hospitalizations occurred in 69% (*n* = 145) of cases, life-threatening events in 11% (*n* = 24), and deaths in 10% (*n* = 21). For skin and subcutaneous tissue disorders, the hospitalization rate was 70% (*n* = 102), with life-threatening events and deaths accounting for 10% (*n* = 15), and 6% (*n* = 9) of cases, respectively. Immune system disorders led to hospitalizations in 53% (*n* = 10) of cases, life-threatening events in 11% (*n* = 2), and deaths in 21% (*n* = 4).

**FIGURE 7:**
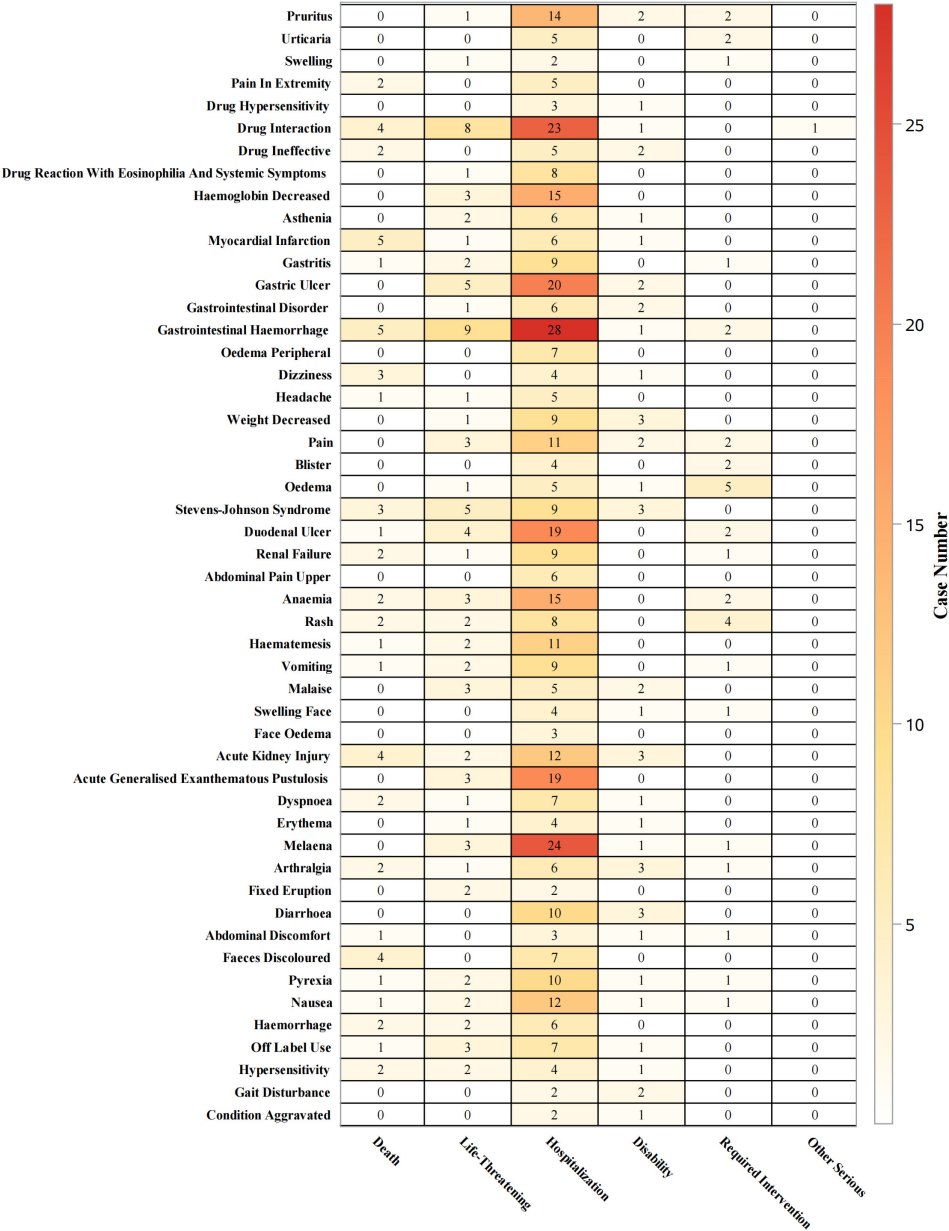
Outcomes by PTs.

At the PT level (Figure 7), gastrointestinal hemorrhage was associated with hospitalizations in 62% (*n* = 28) of cases, life-threatening events in 20% (*n* = 9), and deaths in 11% (*n* = 5). Gastric ulcer led to hospitalizations in 74% (*n* = 20) of cases, life-threatening events in 19% (*n* = 5), and no deaths. Pruritus led to hospitalizations in 74% (*n* = 14), with one life-threatening case (5%) and no deaths. AKI was associated with hospitalizations in 57% (*n* = 12) of cases, life-threatening events in 10% (*n* = 2), and deaths in 19% (*n* = 4). Stevens-Johnson syndrome led to hospitalizations in 45% (*n* = 9) of cases, life-threatening events in 25% (*n* = 5), and deaths in 15% (*n* = 3). AGEP resulted in hospitalizations in 86% (*n* = 19) of cases, life-threatening events in 14% (*n* = 3), and no deaths.

## 4 Discussion

For medications such as piroxicam, continuous post-marketing surveillance and prompt reporting of suspected adverse events are vital for evaluating their benefit risk profiles in real-world settings, which are crucial for clinical decision-making (15). This study provides a comprehensive analysis of AEs linked to piroxicam between Q1 2004 and Q4 2024 using data from the FAERS database. This study confirms well-documented AEs–such as hypersensitivity, gastric ulcer, urticaria, and severe adverse reactions, including gastrointestinal hemorrhage and Stevens-Johnson syndrome–and identifies potentially less well-known signals, including AGEP, blister formation, and urinary retention. These findings underscore the importance of vigilant monitoring during both the early and long-term phases of treatment to mitigate serious outcomes on time.

Piroxicam’s gastrointestinal, hepatic, and renal toxicities are primarily attributed to non-selective COX inhibition and oxidative stress (16). Gastrointestinal toxicity, particularly gastric ulceration, arises due to COX-1 inhibition in the gastric mucosa, which suppresses prostaglandin (PG) synthesis (e.g., PGE2), compromising mucosal integrity via acid suppression, mucus production, and mucosal blood flow regulation (17). This effect is compounded by oxidative stress (e.g., elevated Malondialdehyde, depleted Glutathione, and reduced Catalase activity) and mitochondrial dysfunction-driven apoptosis (via caspase-3 upregulation) (18). Hepatic toxicity is characterized by oxidative damage during hepatic metabolism, resulting in glutathione depletion, reduced antioxidant enzyme activity, lipid peroxidation (Malondialdehyde elevation), and histopathological necrosis (19). Renal injury is linked to COX-1-mediated dysregulation of PG-dependent renal hemodynamics overproduction of reactive oxygen species, which activate caspase-3-mediated apoptotic pathways, leading to AKI or chronic kidney injury (20).

This study identified several AEs not currently listed on piroxicam’s label, including AGEP, blister, and urinary retention. These findings possibly originate from distinct pathophysiological mechanisms. AGEP, for instance, is likely driven by a T cell-mediated delayed hypersensitivity reaction (21), where piroxicam may act as a hapten triggering immune activation, leading to neutrophil infiltration and pustule formation (22). Blister formation may reflect epidermal cell damage or epidermal-dermal separation (23), potentially mediated by keratinocyte apoptosis or antibody-driven inflammation. These conditions may be underdiagnosed due to their rarity or misattribution to other causes in clinical trials. Urinary retention, another unlisted AE, may result from COX inhibition leading to decreased PG synthesis. PGs like PGE2 normally promote the relaxation of bladder muscles (24); their reduction can void function. This effect is particularly concerning in elderly males or individuals with pre-existing urological conditions. Early clinical trials may have underrepresented these populations. These findings suggest the need for increased dermal monitoring during dosing due to the risk of progression to severe skin reactions. In addition, it is advisable to assess prostate function and residual bladder volume in at-risk patients before initiating piroxicam.

The time-to-onset analysis revealed that most piroxicam-related AEs occurred within the first month of therapy with a median onset of 7 days. This highlights the need for close clinical observation during the early stages of treatment to enable timely detection and intervention for emerging adverse reactions, thus potentially improving patient outcomes and safety.

The subgroup analysis reveals significant demographic variations in adverse drug reactions associated with piroxicam. Females exhibit a higher susceptibility to hypersensitivity reactions, likely driven by estrogen-mediated immune bias and sex-specific differences in drug metabolism (25). Conversely, males are more prone to gastrointestinal ulcers and AKI, potentially due to androgen- associated mucosal vulnerability and enhanced suppression of COX-1 (26). Age-stratified data indicate that older adults (≥65 years) disproportionately experience bleeding and renal complications, possibly due to age-related pharmacokinetic alterations and polypharmacy (27). Middle-aged adults (45–64 years) show concurrent risks of allergic rash and gastrointestinal ulceration, reflecting sustained immune reactivity and frequent NSAID use. Younger adults (18–44 years) mainly experience dermatologic reactions such as rashes and AGEP, likely a consequence of robust immune system responses. Notably, in children under 18, rare but concerning adverse events such as eye pain and blurred vision suggest potential vulnerability to developing neural or ocular structures. These findings highlight the need for tailored risk mitigation strategies. Women and younger patients may benefit from early allergy surveillance, while older adults and men may require gastrointestinal and renal protective measures, including prophylactic use of acid-suppressing drugs. Elderly patients, especially those with a history of ulcers or renal impairment, should be screened for latent bleeding tendencies or declining renal function. Ultimately, these data reinforce that a one-size-fits-all dosing approach is inadequate–demographic characteristics must guide piroxicam prescribing decisions.

It is noteworthy that only 1,198 reports were submitted for piroxicam, a drug that has been on the market for many years. We posit that this relatively low number of reports may be attributed to two primary factors. First, the evolution of NSAIDs. Over the past two decades, numerous novel NSAIDs with improved safety profiles have been introduced. Concurrently, well-documented gastrointestinal and cardiovascular safety concerns associated with piroxicam have prompted regulatory warnings, leading to a decline in physician prescribing. Second, underreporting of adverse events. Healthcare providers, patients, and pharmacists may exhibit limited awareness of the FAERS, encounter challenges in attributing adverse events in polypharmacy settings, or perceive mild adverse events as clinically insignificant and therefore not warranting reporting. The events reported in this study may predominantly be severe or rare cases, which, while highlighting significant safety signals, could also underestimate the true frequency and diversity of AEs associated with piroxicam. Future research should integrate additional data sources–such as large-scale observational studies–to provide a more comprehensive assessment of piroxicam’s safety profile and enhance the generalizability of findings.

Like all studies based on the FAERS database, this analysis is subject to inherent limitations. First, FAERS relies on spontaneous, voluntary reporting from multiple sources, often resulting in incomplete, inconsistent, or inaccurate data, which may introduce bias in the analysis. Known AEs associated with piroxicam are more likely to be reported compared to less well-known ones. The Weber effect also plays a role, where the relative change in the perception of an AE’s significance affects reporting. Minor changes in the occurrence of AEs might not be reported if they are not perceived as significant enough relative to the baseline. Media influence can also have a profound impact. Second, critical confounding variables–such as dosage, treatment duration, patient comorbidities, concomitant medications, and other variables potentially influencing AEs–are frequently missing or poorly documented, limiting the ability to control for influencing factors. Third, a fundamental limitation of using the FAERS database is the lack of a denominator. Without knowing the total number of patients exposed to piroxicam, we cannot calculate the absolute risk of any AE. While our disproportionality analysis may show a strong signal, the actual risk to an individual patient could be very low. This lack of absolute risk information limits the clinical utility of our findings. Fourth, the high proportion of “not specified” values for age and sex is a major source of potential bias, especially in subgroup analyses. Certain AEs might be more prevalent in specific age brackets or among a particular sex, but without complete data, we may under- or over-estimate these differences. Fifth, although the method for removing duplicates in the FAERS database is standard, it is imperfect. There is a possibility that some duplicates may remain in our dataset, which can inflate the reported frequencies of AEs and lead to over estimation of the drug’s risk. Sixth, due to the inability to completely eliminate unrecognized deletions in FAERS, the median onset time of 7 days may still slightly underestimate the true value. Finally, the disproportionality analysis conducted here could identify statistical associations but failed to establish causality between piroxicam and the reported AEs. It is crucial to note that the observed signals might be confounded by various factors. For instance, confounding by indication could be at play, where the reason for prescribing piroxicam (such as the patient’s underlying condition) might be the actual cause of the AEs rather than the drug itself. Co-medications are another potential confounder; patients taking piroxicam often use other drugs simultaneously, and the interactions between these drugs could lead to the reported AEs. Underlying diseases can also contribute to this confounding. It serves to generate hypotheses rather than quantify the absolute risk or prove cause-effect relationships. Nevertheless, despite these limitations, the large and diverse pool of international cases in FAERS offers valuable insights and enables the preliminary identification of potential safety signals related to piroxicam.

## 5 Conclusion

This study employed the FAERS database to conduct a thorough and systematic evaluation examination of AEs associated with piroxicam from 2004 through 2024. The analysis confirmed several known safety concerns while uncovering previously unlisted but clinically significant AEs–including AGEP, urinary retention, and blistering. These findings underscore important safety considerations for piroxicam’s clinical use and emphasize the significance of vigilant patient monitoring throughout treatment. Given the drug’s risk profile, sustained pharmacovigilance is essential to ensure the safe administration of piroxicam in routine healthcare practice.

## Data Availability

The original contributions presented in this study are included in this article/Supplementary material, further inquiries can be directed to the corresponding author.

## References

[1] Brogden R Heel R Speight T Avery G. Piroxicam: a review of its pharmacological properties and therapeutic efficacy. *Drugs.* (1981) 22:165–87. 10.2165/00003495-198122030-00001 7021122

[2] Wilk M Zimba O Haugeberg G Korkosz M. Pain catastrophizing in rheumatic diseases: prevalence, origin, and implications. *Rheumatol Int.* (2024) 44:985–1002. 10.1007/s00296-024-05583-8 38609656 PMC11108955

[3] Zhou Y Luo X Li P Liu X Li J Su L The burden of rheumatoid arthritis in China from 1990 to 2019 and projections to 2030. *Public Health.* (2025) 242:71–8. 10.1016/j.puhe.2025.02.033 40037154

[4] Moreira V Signorelli F Hattori W Dionisio V. Association between psychological factors and physical performance in individuals with knee osteoarthritis: A cross-sectional study. *J Bodyw Mov Ther.* (2025) 42:274–82. 10.1016/j.jbmt.2024.12.030 40325680

[5] Khalil N Ahmed E Tharwat T Mahmoud Z. NSAIDs between past and present; a long journey towards an ideal COX-2 inhibitor lead. *RSC Adv.* (2024) 14:30647–61. 10.1039/d4ra04686b 39324041 PMC11423417

[6] Richardson C Blocka K Ross S Verbeeck R. Effects of age and sex on piroxicam disposition. *Clin Pharmacol Ther.* (1985) 37:13–8. 10.1038/clpt.1985.4 3965234

[7] Muthuluri T Chandrupatla S Rajan R Reddy V Jhawar D Potturi A. Pre-emptive analgesia efficacy of piroxicam versus tramadol in oral surgery. *J Dent Anesth Pain Med.* (2022) 22:443–50. 10.17245/jdapm.2022.22.6.443 36601129 PMC9763819

[8] Arellano F Yood M Wentworth C Oliveria S Rivero E Verma A Use of cyclo-oxygenase 2 inhibitors (COX-2) and prescription non-steroidal anti-inflammatory drugs (NSAIDS) in UK and USA populations. Implications for COX-2 cardiovascular profile. *Pharmacoepidemiol Drug Saf.* (2006) 15:861–72. 10.1002/pds.1343 17086563

[9] McGettigan P Henry D. Cardiovascular risk and inhibition of cyclooxygenase: a systematic review of the observational studies of selective and nonselective inhibitors of cyclooxygenase 2. *JAMA.* (2006) 296:1633–44. 10.1001/jama.296.13.jrv60011 16968831

[10] Xu X Guo Q Li Y Zhai C Mao Y Zhang Y Assessing the real-world safety of regadenoson for myocardial perfusion imaging: insights from a comprehensive analysis of FAERS data. *J Clin Med.* (2025) 14:1860. 10.3390/jcm14061860 40142667 PMC11943247

[11] Rothman K Lanes S Sacks S. The reporting odds ratio and its advantages over the proportional reporting ratio. *Pharmacoepidemiol Drug Saf.* (2004) 13:519–23. 10.1002/pds.1001 15317031

[12] Evans S Waller P Davis S. Use of proportional reporting ratios (PRRs) for signal generation from spontaneous adverse drug reaction reports. *Pharmacoepidemiol Drug Saf.* (2001) 10:483–6. 10.1002/pds.677 11828828

[13] Ang P Chen Z Chan C Tai B. Data mining spontaneous adverse drug event reports for safety signals in Singapore – a comparison of three different disproportionality measures. *Expert Opin Drug Saf.* (2016) 15:583–90. 10.1517/14740338.2016.1167184 26996192

[14] Rivkees S Szarfman A. Dissimilar hepatotoxicity profiles of propylthiouracil and methimazole in children. *J Clin Endocrinol Metab.* (2010) 95:3260–7. 10.1210/jc.2009-2546 20427502

[15] Zhao B Zhang X Chen M Wang Y. A real-world data analysis of acetylsalicylic acid in FDA adverse event reporting system (FAERS) database. *Expert Opin Drug Metab Toxicol.* (2023) 19:381–7. 10.1080/17425255.2023.2235267 37421631

[16] Yunita M Plumeriastuti H Agustono B Fahlefi M Fadilla A Pangastutie R The protective effects of garlic (*Allium sativum* var. Solo Garlic) extract mitigates piroxicam-induced liver damage and oxidative stress in rats. *Open Vet J.* (2025) 15:1349–57. 10.5455/OVJ.2025.v15.i3.26 40276184 PMC12017719

[17] Takeuchi K Amagase K. Roles of cyclooxygenase, prostaglandin E2 and EP receptors in mucosal protection and ulcer healing in the gastrointestinal tract. *Curr Pharm Des.* (2018) 24:2002–11. 10.2174/1381612824666180629111227 29956615

[18] Abdeen A Aboubakr M Elgazzar D Abdo M Abdelkader A Ibrahim S Rosuvastatin attenuates piroxicam-mediated gastric ulceration and hepato-renal toxicity in rats. *Biomed Pharmacother.* (2019) 110:895–905. 10.1016/j.biopha.2018.11.004 30572194

[19] Badawi M. Histological study of the protective role of ginger on piroxicam-induced liver toxicity in mice. *J Chin Med Assoc.* (2019) 82:11–8. 10.1016/j.jcma.2018.06.006 30839397

[20] Ebaid H Dkhil M Danfour M Tohamy A Gabry M. Piroxicam-induced hepatic and renal histopathological changes in mice. *Libyan J Med.* (2007) 2:82–9. 10.4176/070130 21503258 PMC3078278

[21] Hadavand M Kaffenberger B Cartron A Trinidad J. Clinical presentation and management of atypical and recalcitrant acute generalized Exanthematous pustulosis. *J Am Acad Dermatol.* (2022) 87:632–9. 10.1016/j.jaad.2020.09.024 32926975

[22] Fernando S. Acute generalised exanthematous pustulosis. *Australas J Dermatol.* (2012) 53:87–92. 10.1111/j.1440-0960.2011.00845.x 22571555

[23] Yang M Wu H Zhao M Chang C Lu Q. The pathogenesis of bullous skin diseases. *J Transl Autoimmun.* (2019) 2:100014. 10.1016/j.jtauto.2019.100014 32743502 PMC7388362

[24] Sellers D Chess-Williams R Michel M. Modulation of lower urinary tract smooth muscle contraction and relaxation by the urothelium. *Naunyn Schmiedebergs Arch Pharmacol.* (2018) 391:675–94. 10.1007/s00210-018-1510-8 29808232

[25] Trenti A Tedesco S Boscaro C Trevisi L Bolego C Cignarella A. Estrogen, angiogenesis, immunity and cell metabolism: solving the puzzle. *Int J Mol Sci.* (2018) 19:859. 10.3390/ijms19030859 29543707 PMC5877720

[26] Lin X Wang W. Analysis of high risk factors for chronic atrophic gastritis. *Saudi J Gastroenterol.* (2023) 29:127–34. 10.4103/sjg.sjg_383_22 36588366 PMC10270474

[27] Demeester C Robins D Edwina A Tournoy J Augustijns P Ince I Physiologically based pharmacokinetic (PBPK) modelling of oral drug absorption in older adults – an AGePOP review. *Eur J Pharm Sci.* (2023) 188:106496. 10.1016/j.ejps.2023.106496 37329924

