# Supplementary material for: A real-world data analysis of piroxicam in the FDA Adverse Event Reporting System (FAERS) database
Source: Front Med (Lausanne). 2025 Oct 10;12:1687088. doi: 10.3389/fmed.2025.1687088 (PMC12549246; doi:10.3389/fmed.2025.1687088)
Supplement: Supplementary file 1 [file Table_1.docx]

Supplementary Material
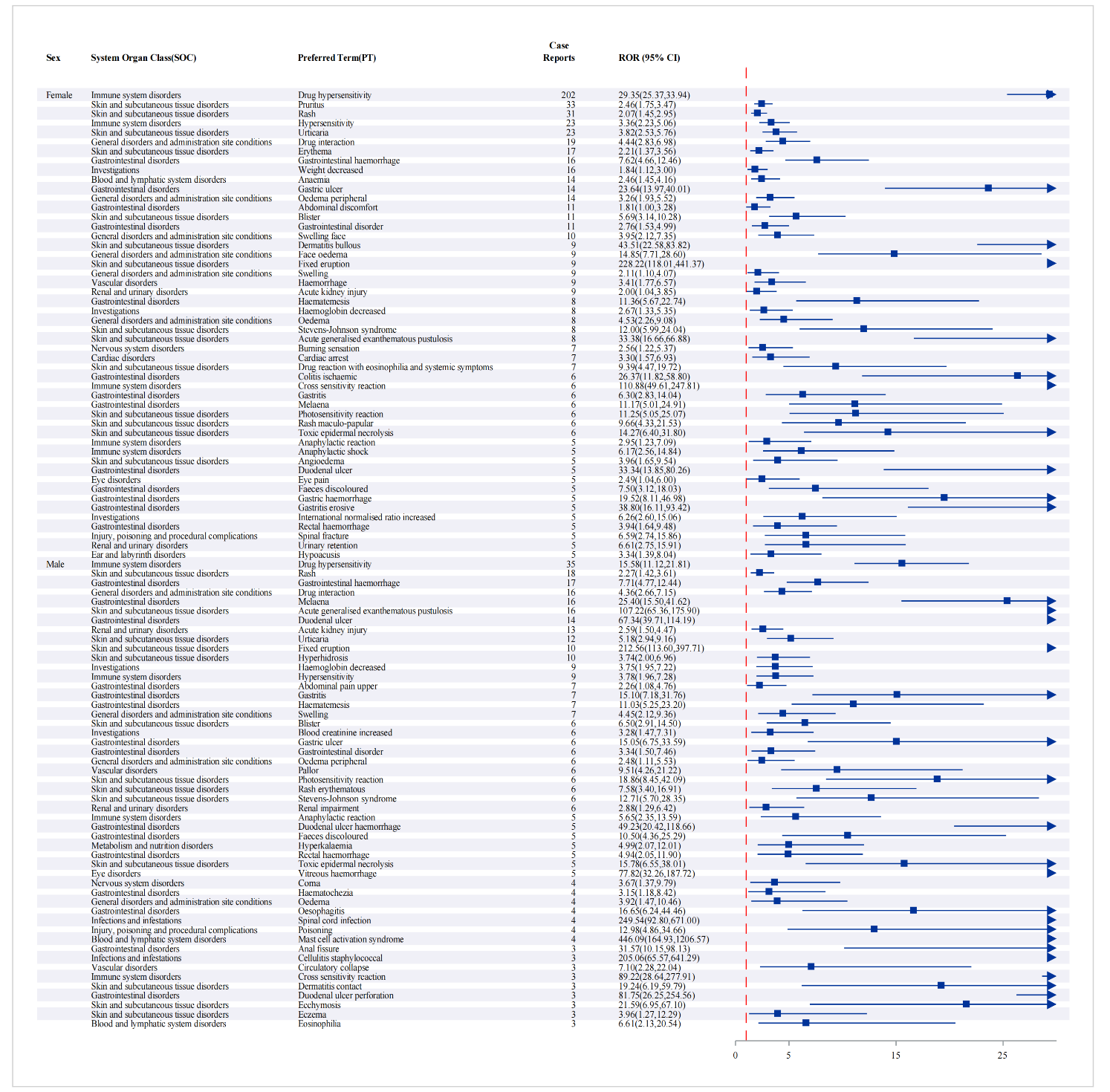


**Supplementary Figure 1.** Gender differences analysis of AEs related to Piroxicam.

**
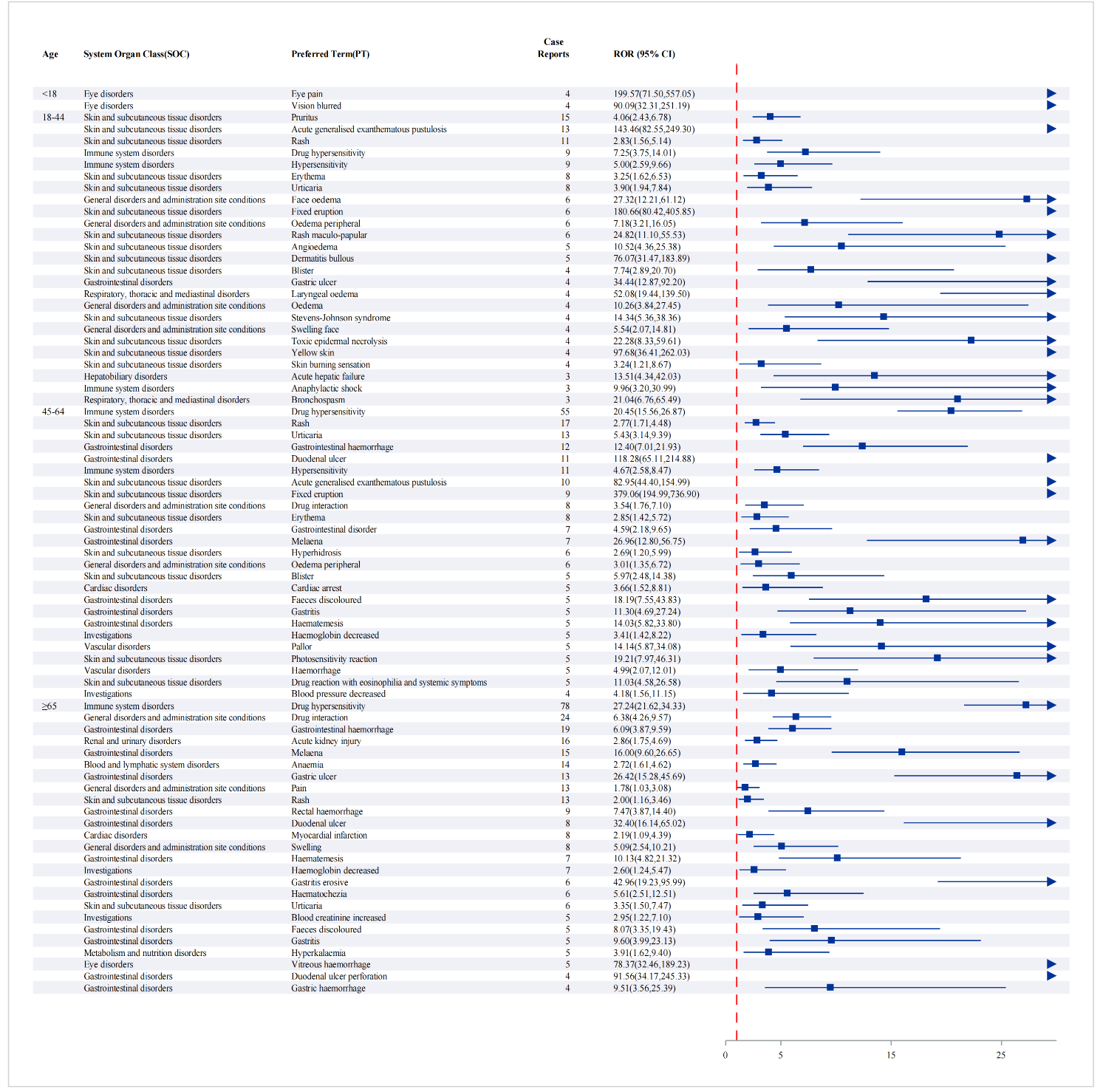
**

**Supplementary Figure 2.** Analysis of age differences in AEs related to Piroxicam.

**
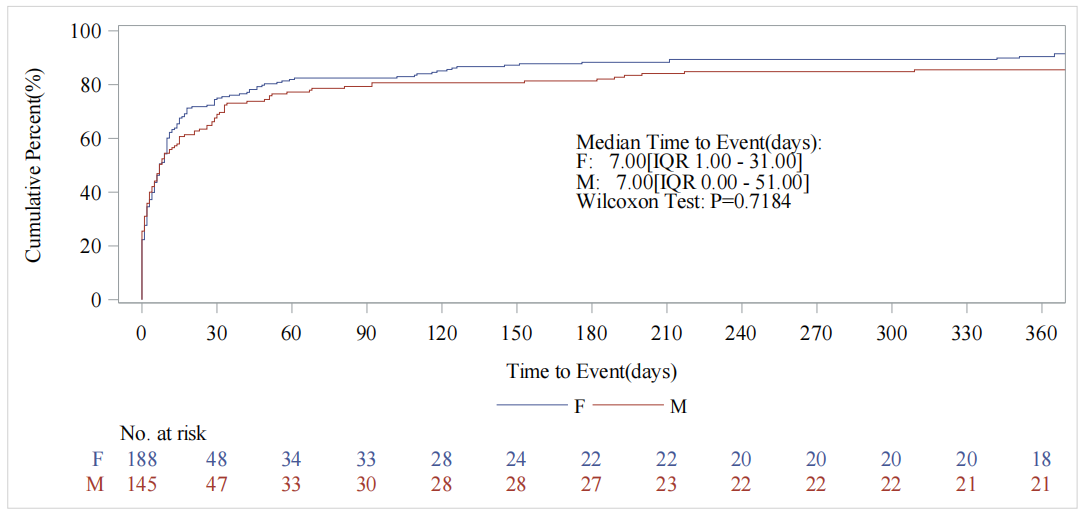
**

**Supplementary Figure 3.** Analysis of gender differences in the occurrence time of AEs related to Piroxicam.

**Supplementary Table 1:** Two-by-two contingency table for disproportionality analyses.

|  | **Target AEs** | **Other AEs** | **Total** |
| --- | --- | --- | --- |
| Piroxicam | a | b | a+b |
| Other drugs | c | d | c+d |
| Total | a+c | b+d | a+b+c+d |

Abbreviations: AEs, adverse events; a, the number of reports containing target AEs caused by Piroxicam; b, the number of reports containing other AEs caused by Piroxicam; c, the number of reports containing target AEs caused by other drugs; d, the number of reports containing other AEs caused by other drugs.

**Supplementary Table 2:** Four primary algorithms used for signal detection.

| Piroxicam | Equation | Criteria |
| --- | --- | --- |
| ROR | 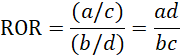  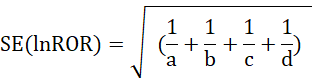  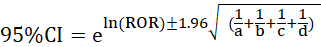 | N ≥ 3 and the 95% CI lower limit > 1 |
| PRR | 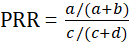  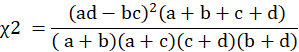 | PRR ≥ 2, χ^2^ ≥ 4, N ≥ 3 |
| BCPNN | IC = 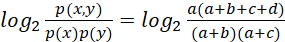  E(IC) = 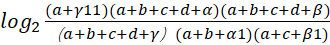  V(IC) = 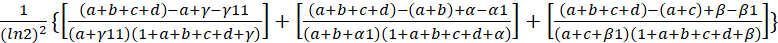  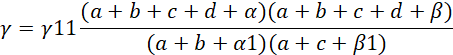  IC-2SD = E(IC)-2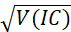  α1 = β1, α = β = 2 | Lower limit of IC025 > 0 |
| MGPS | 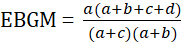  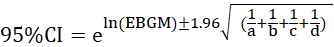 | EBGM05 > 2 |

Abbreviations: AEs, adverse events; a, the number of reports containing target AEs caused by Piroxicam; b, the number of reports containing other AEs caused by Piroxicam; c, the number of reports containing target AEs caused by other drugs; d, the number of reports containing other AEs caused by other drugs; CI, confidence interval; N, the number of reports; χ2, chi-squared; IC, information component; IC025, the lower limit of 95% CI of the IC; E(IC), the IC expectations; V(IC), the variance of IC; EBGM, empirical Bayesian geometric mean; EBGM05, the lower limit of 95% CI of EBGM.

**Supplementary Table 3:** All compliant AEs for Piroxicam at PT levels.

| **Preferred Term (PT)** | **Case reports** | **ROR (95% CI)** | **PRR (χ²)** | **IC (IC025)** | **EBGM (EBGM05)** |
| --- | --- | --- | --- | --- | --- |
| Drug hypersensitivity | 249 | 24.72(21.73-28.13) | 22.99(5245.55) | 4.52(4.21) | 22.95(20.17) |
| Gastrointestinal haemorrhage | 41 | 8.58(6.31-11.68) | 8.49(271.22) | 3.09(2.40) | 8.49(6.24) |
| Drug interaction | 38 | 4.38(3.18-6.03) | 4.34(97.84) | 2.12(1.53) | 4.34(3.15) |
| Urticaria | 36 | 4.07(2.93-5.65) | 4.04(82.48) | 2.01(1.42) | 4.04(2.91) |
| Hypersensitivity | 33 | 3.28(2.33-4.63) | 3.26(51.91) | 1.71(1.11) | 3.26(2.31) |
| Acute generalised exanthematous pustulosis | 27 | 64.73(44.29-94.60) | 64.22(1673.95) | 6.00(3.75) | 63.97(43.77) |
| Melaena | 24 | 19.46(13.02-29.08) | 19.33(416.86) | 4.27(2.90) | 19.31(12.92) |
| Gastric ulcer | 23 | 21.68(14.38-32.68) | 21.54(450.05) | 4.43(2.94) | 21.51(14.27) |
| Fixed eruption | 21 | 223.07(144.83-343.57) | 221.69(4551.89) | 7.77(3.71) | 218.73(142.01) |
| Duodenal ulcer | 20 | 51.53(33.18-80.03) | 51.23(982.08) | 5.67(3.28) | 51.08(32.89) |
| Blister | 17 | 5.72(3.55-9.21) | 5.70(65.84) | 2.51(1.49) | 5.69(3.53) |
| Haematemesis | 16 | 11.28(6.90-18.44) | 11.24(149.15) | 3.49(2.11) | 11.23(6.87) |
| Swelling face | 16 | 4.49(2.75-7.34) | 4.48(43.22) | 2.16(1.19) | 4.47(2.74) |
| Drug reaction with eosinophilia and systemic symptoms | 15 | 9.78(5.89-16.24) | 9.74(117.60) | 3.28(1.93) | 9.73(5.86) |
| Stevens-Johnson syndrome | 14 | 11.25(6.65-19.01) | 11.20(130.05) | 3.48(1.99) | 11.20(6.62) |
| Faeces discoloured | 13 | 10.79(6.26-18.60) | 10.75(114.92) | 3.43(1.89) | 10.74(6.23) |
| Gastritis | 13 | 8.81(5.11-15.18) | 8.78(89.57) | 3.13(1.72) | 8.77(5.09) |
| Oedema | 13 | 4.38(2.54-7.55) | 4.36(33.73) | 2.13(1.04) | 4.36(2.53) |
| Face oedema | 12 | 12.62(7.16-22.25) | 12.58(127.84) | 3.65(1.93) | 12.57(7.13) |
| Photosensitivity reaction | 12 | 13.18(7.47-23.23) | 13.14(134.46) | 3.71(1.96) | 13.13(7.44) |
| Toxic epidermal necrolysis | 12 | 14.80(8.40-26.10) | 14.75(153.76) | 3.88(2.04) | 14.74(8.36) |
| Dermatitis bullous | 11 | 28.02(15.49-50.66) | 27.93(285.15) | 4.80(2.27) | 27.88(15.42) |
| Rectal haemorrhage | 10 | 4.16(2.24-7.75) | 4.16(23.97) | 2.05(0.82) | 4.15(2.23) |
| Cross sensitivity reaction | 9 | 90.01(46.71-173.46) | 89.78(785.80) | 6.48(2.27) | 89.29(46.34) |
| Urinary retention | 9 | 4.93(2.56-9.48) | 4.92(28.07) | 2.30(0.91) | 4.91(2.55) |
| Gastric haemorrhage | 8 | 12.04(6.01-24.09) | 12.01(80.71) | 3.59(1.47) | 12.00(6.00) |
| Gastritis erosive | 8 | 30.15(15.06-60.37) | 30.08(224.51) | 4.91(1.86) | 30.03(14.99) |
| Anaphylactic shock | 7 | 5.12(2.44-10.75) | 5.11(23.17) | 2.35(0.73) | 5.11(2.44) |
| Rash maculo-papular | 7 | 5.86(2.79-12.30) | 5.85(28.12) | 2.55(0.84) | 5.84(2.78) |
| Colitis ischaemic | 6 | 16.59(7.45-36.97) | 16.56(87.67) | 4.05(1.27) | 16.55(7.43) |
| Duodenal ulcer haemorrhage | 6 | 36.79(16.50-82.02) | 36.73(208.05) | 5.20(1.50) | 36.64(16.44) |
| Erythema multiforme | 6 | 11.64(5.22-25.94) | 11.62(58.22) | 3.54(1.11) | 11.61(5.21) |
| Leukocytosis | 6 | 6.06(2.72-13.50) | 6.05(25.29) | 2.60(0.72) | 6.05(2.71) |
| Mouth ulceration | 6 | 5.29(2.37-11.78) | 5.28(20.82) | 2.40(0.62) | 5.28(2.37) |
| Peptic ulcer haemorrhage | 6 | 158.27(70.78-353.92) | 157.99(927.03) | 7.29(1.66) | 156.49(69.98) |
| Vitreous haemorrhage | 6 | 39.49(17.71-88.04) | 39.42(224.13) | 5.30(1.51) | 39.33(17.64) |
| Duodenal ulcer perforation | 5 | 17.29(7.19-41.60) | 17.27(76.57) | 4.11(1.04) | 17.25(7.17) |
| Eyelid oedema | 5 | 7.37(3.06-17.71) | 7.36(27.45) | 2.88(0.66) | 7.35(3.06) |
| Laryngeal oedema | 5 | 14.62(6.08-35.16) | 14.60(63.29) | 3.87(0.98) | 14.59(6.07) |
| Scar | 5 | 5.21(2.17-12.52) | 5.20(16.96) | 2.38(0.43) | 5.20(2.16) |
| Self-medication | 5 | 22.21(9.23-53.44) | 22.18(101.01) | 4.47(1.11) | 22.15(9.21) |
| Acute hepatic failure | 4 | 5.60(2.10-14.94) | 5.60(15.11) | 2.48(0.25) | 5.60(2.10) |
| Cerebellar syndrome | 4 | 32.20(12.07-85.93) | 32.17(120.55) | 5.00(0.86) | 32.10(12.03) |
| Diverticulum | 4 | 9.16(3.43-24.42) | 9.15(29.02) | 3.19(0.51) | 9.14(3.43) |
| Gastric ulcer haemorrhage | 4 | 12.39(4.65-33.05) | 12.38(41.81) | 3.63(0.63) | 12.37(4.64) |
| Gastrointestinal pain | 4 | 6.86(2.57-18.28) | 6.85(19.98) | 2.78(0.37) | 6.85(2.57) |
| Injection site atrophy | 4 | 42.61(15.96-113.74) | 42.56(161.91) | 5.41(0.90) | 42.45(15.90) |
| Oesophageal ulcer | 4 | 18.42(6.90-49.13) | 18.40(65.74) | 4.20(0.75) | 18.38(6.89) |
| Oesophagitis | 4 | 6.63(2.48-17.67) | 6.62(19.07) | 2.73(0.35) | 6.62(2.48) |
| Yellow skin | 4 | 15.28(5.73-40.76) | 15.27(53.28) | 3.93(0.69) | 15.25(5.72) |
